# Gene discovery from *Jatropha curcas *by sequencing of ESTs from normalized and full-length enriched cDNA library from developing seeds

**DOI:** 10.1186/1471-2164-11-606

**Published:** 2010-10-27

**Authors:** Purushothaman Natarajan, Deepa Kanagasabapathy, Gnanasekaran Gunadayalan, Jasintha Panchalingam, Noopur shree, Priyanka Annabel Sugantham, Kavita Kumari Singh, Parani Madasamy

**Affiliations:** 1 Genomics Laboratory, Department of Genetic Engineering, SRM University, Chennai, Tamil Nadu, 603 203 India

## Abstract

**Background:**

*Jatropha curcas *L. is promoted as an important non-edible biodiesel crop worldwide. Jatropha oil, which is a triacylglycerol, can be directly blended with petro-diesel or transesterified with methanol and used as biodiesel. Genetic improvement in jatropha is needed to increase the seed yield, oil content, drought and pest resistance, and to modify oil composition so that it becomes a technically and economically preferred source for biodiesel production. However, genetic improvement efforts in jatropha could not take advantage of genetic engineering methods due to lack of cloned genes from this species. To overcome this hurdle, the current gene discovery project was initiated with an objective of isolating as many functional genes as possible from *J. curcas *by large scale sequencing of expressed sequence tags (ESTs).

**Results:**

A normalized and full-length enriched cDNA library was constructed from developing seeds *of J. curcas*. The cDNA library contained about 1 × 10^6 ^clones and average insert size of the clones was 2.1 kb. Totally 12,084 ESTs were sequenced to average high quality read length of 576 bp. Contig analysis revealed 2258 contigs and 4751 singletons. Contig size ranged from 2-23 and there were 7333 ESTs in the contigs. This resulted in 7009 unigenes which were annotated by BLASTX. It showed 3982 unigenes with significant similarity to known genes and 2836 unigenes with significant similarity to genes of unknown, hypothetical and putative proteins. The remaining 191 unigenes which did not show similarity with any genes in the public database may encode for unique genes. Functional classification revealed unigenes related to broad range of cellular, molecular and biological functions. Among the 7009 unigenes, 6233 unigenes were identified to be potential full-length genes.

**Conclusions:**

The high quality normalized cDNA library was constructed from developing seeds of *J. curcas *for the first time and 7009 unigenes coding for diverse biological functions including oil biosynthesis were identified. These genes will serve as invaluable genetic resource for crop improvement in jatropha to make it an ideal and profitable crop for biodiesel production.

## Background

*Jatropha curcas *L., one of the 175 species in genus Jatropha of the family Euphorbiaceae is a perennial small tree or large shrub native to tropical America and is distributed throughout the tropics and subtropics of Asia and Africa [1]. Recently, jatropha oil is promoted as alternative transport fuel which can be directly blended with petro-diesel or transesterified with methanol and used as biodiesel. From a global food security point of view, jatropha being a non-edible crop which can be grown in areas not suitable for agriculture, is a preferred source for biodiesel feedstock as it does not compete with production of food crops. It reduces the dependence on fossil fuel which is often imported by using precious foreign currency. Its decentralized production will provide income for a large number of small and marginal farmers. Biodiesel is also less harmful to the environment in that its production and combustion reduces emission of greenhouse gases by 41% relative to fossil fuel [2]. It is reported that biodiesel emits less particulate matter than diesel upon combustion [3]. In fact, large scale jatropha cultivation will improve the environment by greening the area and transforming the wasteland to productive land by preventing soil erosion, causing accumulation of organic matter, increasing soil microbial activity, etc.

The demand for biodiesel production is very huge that it cannot be met from wild grown plants. Increasing the jatropha production requires both to bring more area under cultivation and to enhance productivity. Though jatropha can grow and survive in wasteland with less water, nutrient, and virtually no pest and disease management, productive growth and better yield under restrictive environmental conditions require the development of resilient genotypes. Genotypes with improved drought tolerance are preferred for plantations in marginal lands. Large scale plantations may bring in new challenges which need to be addressed. It was reported that *J. curcas *planted in continuous stretches as a monocrop were devastated by flower and seed feeding insects *Scutellera nobilis *and *Pempelia morosalis *[4]. This indicates that plant breeding programs to develop pest and disease resistance are required when large scale cultivation of jatropha is planned.

In addition, jatropha oil composition itself may have to be modified to make it the best feedstock for biodiesel production. Oils with more of saturated fatty acids give higher cetane number, and oxidative stability which are desirable for combustion/ignition quality and shelf life of biodiesel, respectively. But jatropha oil contains less of saturated fatty acids (21.6%) and more of unsaturated fatty acids (78.4%) [5]. While viscosity of petro-diesel is 2.6 mm^2^/s, it is 4.8 mm^2^/s for biodiesel derived from jatropha oil. Viscosity affects atomization of the fuel upon injection into the combustion chamber, and thereby, increases the formation of engine deposits [6]. More the carbon number of saturated fatty acids higher will be the viscosity. Jatropha contains 84.5% 18-carbon fatty acids and only 14.9% 16-carbon fatty acids [5]. It is possible to make significant changes in the jatropha oil composition by genetic engineering of the metabolic pathway of oil biosynthesis. Therefore, there is a need and scope for genetic improvement of jatropha by using plant breeding and genetic engineering methods.

Genetic intervention in jatropha requires understanding of the biosynthetic pathways, metabolic flux control points, cloning of the genes that code for the enzymes and proteins involved in the metabolic pathways and development of molecular markers. Molecular studies in jatropha are limited and only a few genes have been isolated from jatropha [7,8]. Currently, the dbEST of NCBI contains only 250 annotated Expressed sequence tags (ESTs) from jatropha. We have initiated a gene discovery project by large scale sequencing of ESTs. ESTs are short, single pass sequence reads from 5'or 3' end of randomly selected cDNA clones. Sequencing of ESTs has been successfully employed in several plants including tomato [9], citrus [10], castor [11], arabidopsis [12], rice [13], water melon [14], radish [15] with the objective of gene discovery, metabolic pathway interpretation, gene cloning, molecular marker development, construction of genetic and physical map and comparative mapping and analysis. When sequencing of ESTs is employed for gene discovery purpose, normalization of the cDNA library will greatly increase the efficiency and economy of the process. Normalization reduces the frequency of abundant genes (hundreds of mRNA copies per cell) and enriches the library with rare genes (< 10 mRNA copies per cell) [16]. Since sequencing of ESTs is carried out with the ultimate objective of cloning the genes, it would be highly desirable to combine the normalization with enrichment for full-length clones. This paper reports construction of a normalized and full-length enriched cDNA library from developing seeds of *J. curcas *and isolation of 7009 unigenes by sequencing of 12,084 ESTs.

## Results and Discussion

### Construction of cDNA Library

Construction of cDNA library and sequencing of ESTs helps in rapid gene discovery especially in non-model organisms where no prior sequencing data is available. Next generation sequencing technologies can circumvent the need for constructing cDNA libraries and generate extraordinarily huge amount of sequencing data to further speed up the gene discovery process. However, sequencing of cDNA clones has several advantages over the next generation sequencing methods such as higher average read length, virtually no assembly problem, ability to isolate full length genes without going for RACE PCR and availability of physical clones for further characterization and applications.

For the present study, a normalized and full-length enriched cDNA library was constructed from developing seeds of *J. curcas*. The normalization efficiency was monitored by using chloramphenicol reporter gene. Before normalization, the reporter gene was added to the cDNA population to a redundant rate of about 1% which was found to be reduced to less than 0.025% after normalization. This indicated 40 fold reduction in abundance due to normalization. This normalization will greatly help to enrich the library for rare genes. In addition, it will increase the rate recovery of unigenes and reduce the cost of sequencing by avoiding redundant clones. In fact, the rate of recovery of unigenes in this study was about 58% (7009 unigenes out of 12,084 ESTs) which is much higher than 30 to 40% reported from non-normalized cDNA libraries [11,17,18].

**Figure 1:**
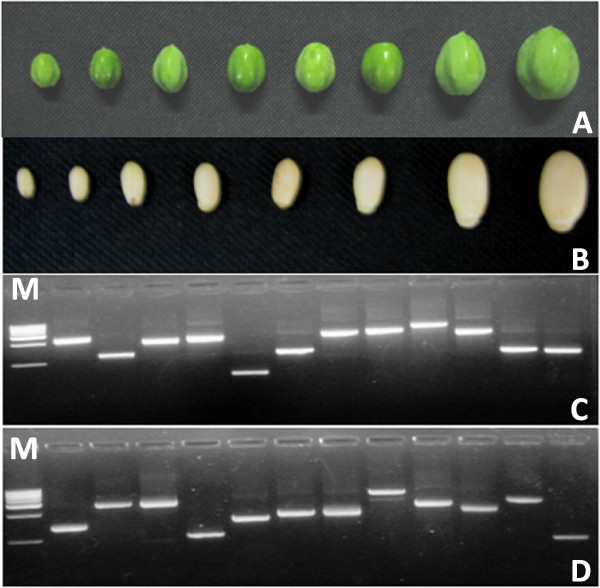
**Construction of cDNA library from *J. curcas***. (A) Fruits of *J. curcas *in different sizes and green in colour (A) and the seeds were removed for total RNA isolation (B). Average insert size of the cDNA library was determined by colony PCR analysis of randomly selected clones(C & D). 1.0 kb ladder DNA size markers (lane M) were used for size determination.

The cDNA library can be more efficiently used for gene discovery if normalization is combined with enrichment for full-length genes. This was done by removing cDNAs smaller than 0.5 kb. Removal of smaller fragments will also increase the cloning efficiency of longer cDNAs. The cDNA library constructed for this study was estimated to contain about 1 × 10^6 ^clones. Ninety six clones were randomly selected to test for the enrichment of full-length genes in these clones. The insert size ranged between 0.8 kb and 3.2 kb with an estimated average insert size of 2.1 kb (Figure 1). BLASTX analysis of the sequences revealed that 94% of the clones could potentially encode for full-length genes. These results indicated that this cDNA library could be reliably used for the gene discovery project in jatropha.

### Sequencing of Expressed Sequence Tags (ESTs)

From the normalized and full-length enriched cDNA library, 13,220 clones were patched for plasmid DNA isolation. Clones which did not grow in the selection medium or did not yield sufficient quantity or quality of plasmid DNA were discarded. Plasmid DNA suitable for sequencing was isolated from 12,810 clones. These clones were sequenced using M13 reverse primer (CAGGAAACAGCTATGAC) which directly reads the cDNAs from the 5' end. Nucleotide bases having Phred value less than 20 were discarded. Vector backbone and additional sequences that were added during cDNA synthesis were removed. After the trimming exercise 144 empty clones (resulted in 0 bases after trimming) and 12,084 high quality ESTs were obtained. The read length of the high quality ESTs ranged between 105 bp and 874 bp with an average length of 576 bp which is comparable with other reports [19,20,18].

### Contig Assembly of ESTs

**Figure 2:**
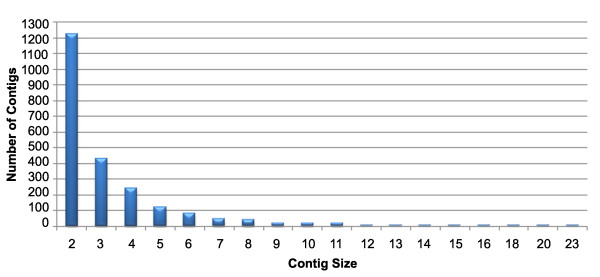
**Distributions of ESTs in Contigs**. Assembly of 12,084 ESTs resulted in 2258 contigs comprising 7333 ESTs. The distribution of 7333 ESTs in each contig was ranged between 2 and 23. The contig size represents the number of ESTs in the contig.

Contig assembly of the 12, 084 ESTs was done to remove the redundant ESTs so that the unique ESTs (unigenes) can be annotated. The summary of the contig assembly is given in Table 1. It showed 2258 contigs and 4751 singletons. The contig size ranged between 2 and 23 (Figure 2) and there were 7333 ESTs in the 2258 contigs indicating the presence of 5075 redundant ESTs. The 2258 contigs were manually checked and the longest EST from each contig was selected as unigene. These representative ESTs from contigs and the 4751 singletons together resulted in 7009 unigenes from the 12,084 ESTs assembled.

**Table 1:** Summary of Contig assembly

Description	Number	Percentage
Total number of ESTs assembled	12084	**-**
Number of contigs	2258	-
Number of ESTs in contigs	7333	60.6
No. of ETS as singletons	4751	39.3
Number of unique ESTs (unigenes)	7009	58.0

Sequencing of ESTs from developing seeds often showed the abundance of genes coding for seed storage proteins [11,21,22]. On the contrary, the current study the showed the abundance of genes related to stress response, disease resistance and plant development (Table 2). The largest contig contained 23 ESTs coding for phosphoethanolamine N-methyltransferase which is essential for phosphatidylcholine biosynthesis. It is reported that 60.5% phospholipid in jatropha seed is composed of phosphatidylcholine [23]. Phosphatidylcholine is hydrolyzed into phosphatidic acid and choline. Phosphatidic acid acts as second messenger in stress signaling, and choline is a precursor for glycine betaine synthesis. Glycine betaine is a compatible solute and its accumulation is widely reported to confer salt and oxidative stress [24,25]. Other contigs contained ESTs coding for indole-3 acetic acid amido synthetase, ccr4 associated factor, ethylene responsive transcription factor and calcineurin B which are also involved in biotic and abiotic stress responses in plants [26-29]. The contigs also represented ESTs coding for protein disulfide isomerase, copine and sucrose synthase which are involved in seed development [30,31] and seed size [32].

**Table 2:** List of abundant ESTs from the normalized cDNA library of *Jatropha curcas *L

Contig ID	No. of ESTs	Annotation	Percentage*
Contig 1	23	Phosphoethanolamine N-methyltransferase	0.33
Contig 2	20	Conserved hypothetical protein	0.28
Contig 3	20	Phosphoric diester hydrolase	0.28
Contig 4	18	Disease resistance protein RPM1	0.26
Contig 5	18	Protein disulfide isomerase	0.26
Contig 6	18	Indole-3-acetic acid-amido synthetase GH3.3	0.26
Contig 7	18	Hypothetical protein	0.26
Contig 8	16	Hypothetical protein	0.23
Contig 9	16	Receptor protein kinase	0.23
Contig 10	16	Branched-chain amino acid aminotransferase	0.23
Contig 11	15	Copine	0.21
Contig 12	15	5-AMP-activated protein kinase	0.21
Contig 13	15	ATP binding protein	0.21
Contig 14	15	Vacuolar Ca2+/H+ exchanger	0.21
Contig 15	14	AMSH	0.20
Contig 16	14	UDP-glucosyltransferase	0.20
Contig 17	14	UDP-glucuronate 5-epimerase	0.20
Contig 18	14	ccr4-associated factor	0.20
Contig 19	14	Conserved hypothetical protein	0.20
Contig 20	13	5-methyltetrahydropteroyltriglutamate-homocysteine S-methyltransferase	0.19
Contig 21	13	RNA recognition motif (RRM)-containing protein	0.19
Contig 22	13	Serine/threonine-protein kinase ASK1	0.19
Contig 23	13	SEC14 cytosolic factor family protein	0.19
Contig 24	13	Conserved hypothetical protein	0.19
Contig 25	13	Threonyl-tRNA synthetase	0.19
Contig 26	12	Sugar transporter	0.17
Contig 27	12	ARMADILLO REPEAT ONLY 1(ARO1)	0.17
Contig 28	12	Cytochrome P450	0.17
Contig 29	11	DELLA protein	0.16
Contig 30	11	Sucrose synthase	0.16
Contig 31	11	Transferase	0.16
Contig 32	11	Bifunctional dihydrofolate reductase-thymidylate synthase	0.16
Contig 33	11	Ethylene-responsive transcription factor	0.16
Contig 34	11	Calcineurin B	0.16
Contig 35	11	Beta-alanine-pyruvate aminotransferase	0.16
Contig 36	11	Poly(A)-binding protein	0.16
Contig 37	11	Metallothionein	0.16

* calculated as percentage of 7009 unigenes

### Annotation of Unigenes

**Figure 3:**
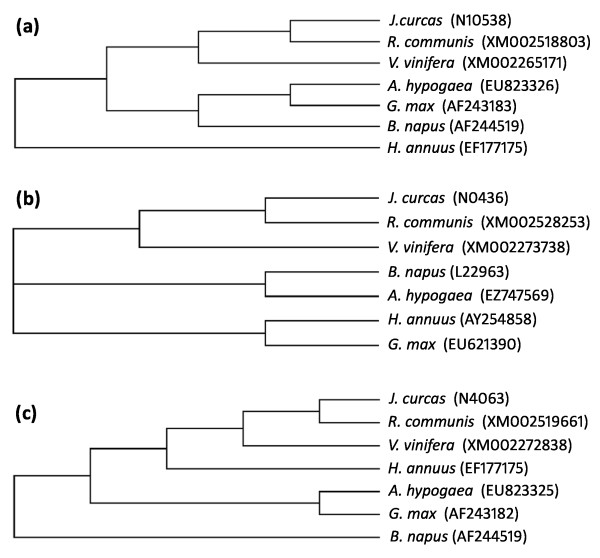
**Phylogenetic analysis**. Phylogenetic trees were generated using the DNA sequences coding for (a) beta-keto acyl ACP synthase II, (b) omega-3-fatty acid desaturase, and (c) beta-keto acyl ACP synthase I from *J. curcas, R. communis, G.max, A. hypogaea, B. napus, H. annuus and V. vinifera*. Clone IDs (for *J. curcas*) and Genbank accession numbers were given in the parentheses.

In total, 3982 unigenes (56.8%) showing significant similarity with genes available in the non-redundant database were identified. Most of these unigenes showed highest similarity with genes from castor (*Ricinus communis)*. This is expected because jatropha itself is called 'wild' castor and both species belong to the family Euphorbiaceae. Next to castor, most of the unigenes showed similarity with genes from grape (*Vitis vinifera*) that belongs to the family Vitaceae. Phylogenetic analysis using 10 selected ESTs by including orthologs from five oilseed crops and *V. vinifera *also revealed that *J. curcas *is closely related to *R. communis *followed by *V. vinifera *(Figure 3, data shown for 3 genes). This association is totally unexpected according to the morphological system of classification [33] in which *Jatropha *belongs to monochlamideae whereas *Vitis *belongs to polypetalae. However, more recent Angiosperm Phylogeny Group Classifications, APGII and APGIII [34,35], which are based on cladistic analysis of larger data sets involving DNA sequences or other forms of systematic data show many contradictory relationships [36]. According to these classifications, the malpighiales (Euphorbiaceae) and Vitales (Vitaceae) are placed much closer under a major core eudicots clade, Rosids. Our data based on coding genes corroborate the APG classification with regard to *Jatropha *and *Vitis*.

### Genes for oil biosynthesis and β-oxidation

Recently jatropha seed oil is widely used for biodiesel production as an alternative renewable energy source. It is important to undertake genetic improvement of this crop to increase oil content, to modify the oil composition, to remove toxic compounds, to increase drought tolerance etc. Seed oil content in brassica and arabidopsis has been increased by the overexpression of diacyl glycerol acyl transferase, lysophosphatidic acid acyltransferase, and glycerol-3-phosphate acyltransferase [37-39]. Seed oil composition has been changed in soybean by using mutant 3-Keto-acyl-ACP synthase II gene which increases the 16-carbon fatty acids and decreases 18-carbon fatty acids [40]. Silencing of stearoyl-ACP desaturase has dramatically increased the content of saturated fatty acid (stearic acid) from 1.2% to 32% in brassica [41]. Therefore, it is possible that increased oil content and specialized seed oil composition can be achieved in jatropha also, provided the genes involved in rate limiting steps of oil biosynthesis pathway are cloned. Important oil biosynthesis genes identified in the present study include the genes involved in fatty acid biosynthesis in plastids (carboxyl transferase of ACCase β subunit, biotin carboxyl carrier protein of ACCase, malonyl-CoA:ACP transacylase, 3-keto acyl ACP reductase, beta-keto acyl ACP synthase I, beta-keto acyl ACP synthase II and acyl carrier protein), desaturation of fatty acids (ω-3-fatty acid desaturase and ω-6-fatty acid desaturase), hydrolysis of fatty acids from acyl-ACP (acyl ACP thioesterase A), activation and transport of free fatty acids to endoplasmic reticulum (long chain acyl-CoA synthetase, acyl -CoA binding protein) and serial incorporation of activated fatty acids to the glycerol backbone to form triacylglycerol or oil (glycerol-3-phosphate acyl transferase, lysophosphatidic acid acyl transferase). Some of these genes are being cloned in plant expression vector for functional evaluation in arabidopsis and tobacco. The unigene collection also contained genes for acyl-CoA oxidase, enoyl-CoA hydratase, β-hydroxyl acyl-CoA dehydrogenase and acyl-CoA acetyl transferase which are involved in β-oxidation of fatty acids and their derivatives. Many of the mutant studies revealed the importance of these enzymes in the breakdown of reserve triacylglycerol, seed development, seed germination, vegetative and reproductive growth phases [42]. Hence the cloning of the genes for beta oxidation pathway will be a valuable source for the genetic manipulation of oil degradation and plant growth.

### Genes for crop improvement

Jatropha is a non-edible plant proposed to be grown in areas not suitable for agriculture such as wasteland, sides of railway track, lands with severe water scarcity, saline areas etc. Hence jatropha should be able to withstand these stresses. Plants respond to these stresses by modulating gene expression, which restores the cellular homeostasis, detoxification and recovery of growth [43,44]. For example, overexpression of betaine aldehyde dehydrogenase gene conferred salt stress tolerance in carrot, maize and tomato [45-47] and Zhang et al. [48] showed that glycine betaine level is increased under drought, heat and salt stresses in jatropha also. When enzymes for glycine betaine biosynthesis are expressed in plants that do not naturally produce glycine betaine, they accumulate little glycine betaine, because their endogenous choline supply is inadequate [49,50]. These plants may require overexpression of phosphoethanolamine N-methyltransferase to overproduce choline. Overexpression of Na+/H+ antiporter from *Pennisetum glaucum *conferred high level of salinity tolerance in transgenic Brassica [51] and overexpression of *E. coli *trehalose-6-phosphate synthase gene conferred drought and salt tolerance in rice [52]. The stress related unigenes identified in the current study include the genes coding for phosphoethanolamine N-methyltransferase, Na+/H+ antiporter, trehalose-6-phosphate synthase, glutathione peroxidase, glutathione s-transferase, spermidine synthase, ethylene-responsive transcription factors, ascorbate peroxidase, late embryogenesis abundant proteins, aquaporin, and salt tolerance protein.

The genes that code for the enzymes involved in different metabolic pathways are very important for genetic manipulation in jatropha. Several genes involved in diverse metabolic pathways such as phospholipids biosynthesis, flavanol synthesis, glycolysis, TCA cycle, HMP shunt, glycogenesis were identified from the current study. Other important genes identified from this study are ferritin, mevalonate kinase, lipoxygenase, glutamate decarboxylase, ent-kaurenoic acid oxidase, cinnamoyl-CoA reductase, zeta-carotene desaturase, gibberellin 2-oxidase, lipoic acid synthetase and beta-carotene hydroxylase. The unigenes also included 208 gene families with 3 or more genes. There were 15 families with more than 10 genes and a highest number of 46 members were identified in serine-threonine kinase gene family. Other gene families included glucan endo-1,3-beta-glucosidase precursor, aspartic proteinase nepenthesin-1 precursor, calmodulin binding protein, F-Box family protein, WD-repeat protein, pentatricopeptide repeat-containing protein, cytochrome P450 etc.

### Genes with unknown functions

BLASTX results showed that 40.4% of *J. curcas *unigenes (2836) are having significant similarity with genes that code for unknown, hypothetical and putative proteins. This is significantly higher than 13 to 25% unknown genes reported in arabidopsis, citrus and oil palm [12,53,54]. It was found that 2.8% of *J. curcas *unigenes (191) did not have significant similarity with any genes available in the non-redundant database at NCBI. This is significantly lower than 8 - 24% of such genes reported in other plants [55,12,56]. These genes are very important as they may be specific to jatropha.

### Full-length unigenes

**Figure 4:**
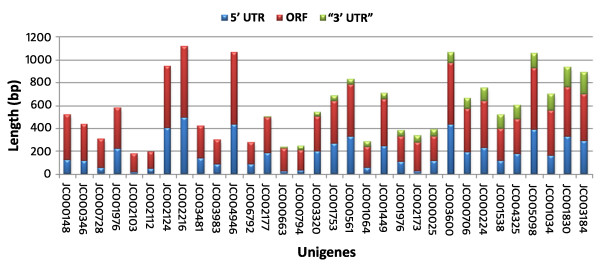
**ORF analysis**. The unigenes that did not show significant similarity to any genes in the protein database at NCBI were taken for ORF prediction using ORF finder tool at NCBI to identify potential full-length unigenes. Putative ORFs were predicted for 32 unigenes and 14 of them were found to be potential full-length unigenes.

Almost all of the unigenes will be full-length at the 3' end because first strand cDNA synthesis was carried out using oliog-dT(15) primer which initiates synthesis from poly (A) tail of the mRNAs. Therefore, full-length nature of the cDNAs at the 5' end was analyzed using the BLASTX results. This could be done for 6818 of the 7009 unigenes which showed significant similarity with genes in the non-redundant database at NCBI. It was found that 91.4% of the unigenes (6233) potentially code for full-length genes. This is significantly higher than 60-75% full-length genes reported before [55,10,57]. The remaining 191 unigenes that could not be analyzed by BLASTX for lack of similarity with genes in the database at NCBI were analyzed by predicting open reading frames (ORFs). In 14 unigenes, 5'UTR and single longest ORF covering almost the entire lengths of the sequences were identified (Figure 4). These unigenes could also be considered as potential full-length unigenes. In 18 unigenes, 5'UTRs were present but the ORFs terminated prematurely. We could not predict ORFs in the remaining 159 unigenes. These results show that the library is highly enriched with full length genes that it can be highly useful for gene discovery purpose.

### Functional Classification

**Figure 5:**
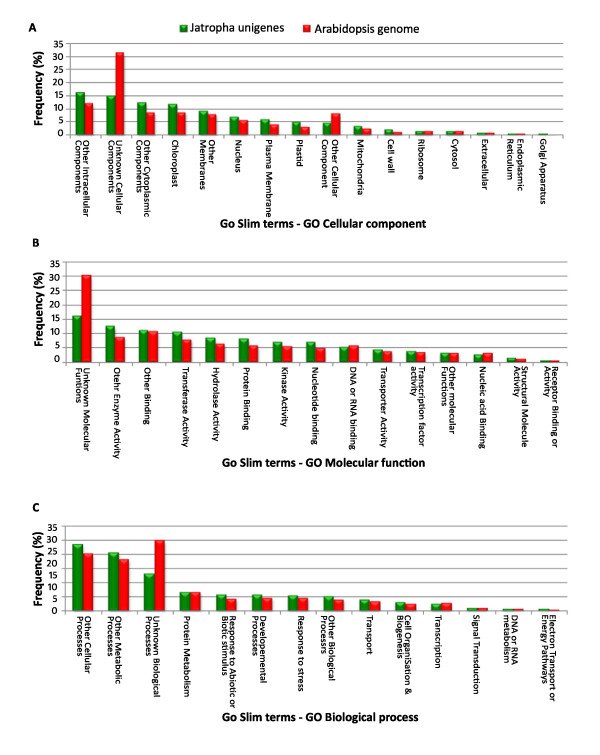
**Functional categorization of unigenes**. GO annotations for 7009 unigenes of Jatropha were retrieved by submitting the list of locus id for the unigenes obtained from BLAST search to the TAIR GO annotation search tool (GO Slim) at The Arabidopsis Information Resource (TAIR). This resulted in the unigenes were functionally classified under three main functional categories: cellular component (A), molecular function (B) and biological process(C) with respective GO Slim terms. The functional coverage of the Jatropha unigenes was also compared with the arabidopsis genome for each category using GO Slim terms.

Functional coverage of the unigenes was identified by comparing the functional distribution of the genes from fully sequenced *A. thaliana *genome. Unigenes were searched against *A. thaliana *genome for functional annotation and locus identifiers using BLASTN at TAIR. The Gene Ontology annotations were assigned for each unigene based on the locus identifiers using GO annotation and categorization tool at TAIR. The 7009 unigenes from *J. curcas *were classified under three broad functional categories using GO slim terms. Distribution of *J. curcas *unigenes and *A. thaliana *genome under these three broad functional categories is shown in Figure 5. This classification provides information on percentage of *J. curcas *unigenes involved in the signal transduction, anabolism, catabolism, reproduction and so on. The results showed that the unigenes cover all the GO slim terms in Arabidopsis.

The unigenes were first classified according their function in 16 different cellular compartments and anatomical structures such as endoplasmic reticulum, plastid, mitochondria, nucleus, cell wall, golgi apparatus etc. which may have unique genes or specific expression profiles. The proportion of genes identified under each class was comparable with *A. thaliana *genome except those unigenes which were classified under 'unknown cellular components' (Figure 5A). Majority of the unigenes were grouped under 'other intra-cellular components', 'unknown cellular components', 'other cytoplasmic components' and 'chloroplast', which accounted for about 55% of the unigenes. Though, non-green tissue was used for the cDNA library construction, about 12% of the unigenes belonged to chloroplast cellular component.

The unigenes were then classified according to their involvement in 14 different biological processes such as protein metabolism, developmental processes, response to stress, transcription, signal transduction etc. These processes are very important for a cell to live and reproduce. Hence, the genes that cover the biological processes are of great importance for functional study. It was found that the larger part of the *J. curcas *unigenes were grouped under 'other cellular process', 'other metabolic process' and 'unknown biological processes' accounting for 23.4%, 20.6% and 13.3%, respectively (Figure 5B).

The unigenes were finally classified under 15 different molecular functions which mainly correspond to the activities performed by the gene products from individual gene or group of genes such as transferase activity, hydrolase activity, kinase activity, receptor binding, receptor activity etc. Greater part of the unigenes was classified under 'unknown molecular function', 'other enzyme activity', 'other binding', 'transferase activity' which accounted for 49.3% of the unigenes (Figure 5C).

### Validation of ESTs

**Figure 6:**
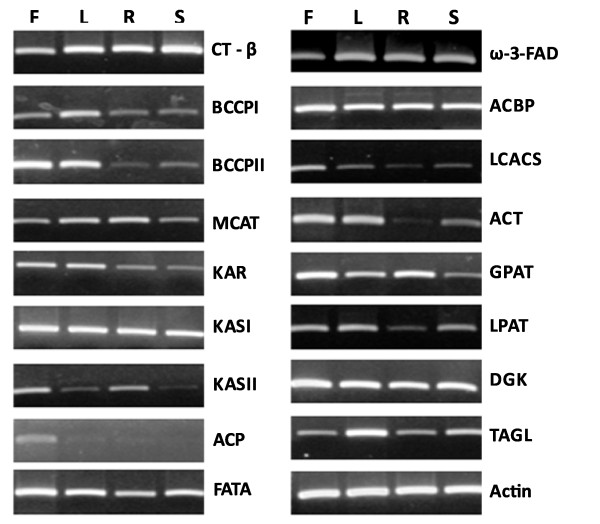
**Semi-quantitative RT-PCR analysis of expression of selected genes**. Expression of seventeen genes involved in oil biosynthesis were analysed by Semi-quantitative RT-PCR in flower (F), mature leaves (L), root (R), and developing seeds (S) of *J. curcas*. Actin was used as an internal control. Name of the genes studied, the primers used for PCR amplification, and the size of the PCR amplified fragments are given in Table 3.

In order to validate the expression of ESTs, a set of 17 ESTs for representing oil biosynthesis genes were selected and their expression was studied in roots, mature leaves, flowers and developing seeds of *J. curcas*. These ESTs were first sequenced from the 3' ends and primers specific to 3'UTR were designed to increase their specificity to the respective transcripts. Gene expression was studied by using semi-quantitative RT-PCR and actin was used as an internal control (Figure 6). The results confirmed that the transcripts representing all the selected ESTs are actively expressed in *J. curcas*. All the transcripts were found to be expressed in all the tissues without significant variation in the level of expression, except ACP gene which showed significantly higher expression in flower. O'Hara et al. [58] have also reported that in flowers, the ACP gene is expressed at higher level than the other genes involved in fatty acid biosynthesis.

## Conclusions

Normalized and full-length enriched cDNA library was constructed and 12,084 clones were sequenced from *J. curcas *for the first time. From these sequences, 7009 unigenes were identified which included 6233 potential full-length genes. These genes encoded for diverse biological functions in *J. curcas *including oil biosynthesis, stress response, flavanol biosynthesis etc. These genes will serve as invaluable resource for the genetic engineering to modify oil composition and to increase oil content, seed yield, pest and disease resistance to make jatropha more suitable for biodiesel production and profitable to farmers. Gene discovery from other tissues of *J. curcas *are being attempted by using next generation sequencing technology.

## Methods

### Collection of Seeds

Seeds in different developmental stages were collected from *Jatropha curcas *fruits which were between 0.5 and 2.5 cm in diameter and green in colour (Figure 1). The seeds were flash frozen in liquid nitrogen and stored at -86°C until further use.

### Isolation of Total RNA

Total RNA from developing seeds was prepared using Trizol reagent following the manufacturer's protocol (Invitrogen, USA). One gram of the developing seeds were ground with liquid nitrogen to a fine powder and suspended in 10 ml Trizol reagent. The suspension was mixed well, and incubated on ice for 10 min. Subsequently, 2 ml chloroform was added to the suspension and incubated on ice for 10 min. The suspension was centrifuged at 10,000 × g for 15 min at 4°C. The aqueous phase was transferred to a fresh tube, and equal volume of isopropanol was added. It was incubated at -20°C for 1 h, and total RNA was recovered by centrifugation at 10,000 × g for 20 min at 4°C. The supernatant was discarded and the pellet was washed with 70% ethanol, dried and dissolved in 200 μl RNase free water. The total RNA was further purified by using RNeasy Mini Kit following the manufacturer's protocol (Qiagen, Hilden, Germany). Quality of total RNA was checked by agarose gel electrophoresis as well as OD_260_/OD_280 _ratio before using it for cDNA synthesis.

### cDNA Synthesis and Normalization

Synthesis of full length cDNAs from total RNA and normalization were performed as described by Patanjali et al. [59] and Soares et al. [60] with slight modifications. Two cDNA preparations were made using the same total RNA. One was used as tester cDNA and the other was used as driver cDNA. First strand tester cDNA and driver cDNA were synthesized using tester3 primer (CAGTGGTATCAACGCAGAGTGGCCGAGGCGGCCT_15_) and driver3 primer (GGGATAACAGGGTAATGGCCGAGGCGGCCGACATGT_15_), respectively. Tester adaptor (GTAACTAGGCCGTAATGGCCACTCTGCGTTGATACCACTG) and driver adaptor (GGCCGTAATGGCCTCGCTACCTTAGGA) were ligated to the 3'end of the newly synthesised first strand tester cDNA and driver cDNA, respectively. These adaptors will ligate only to the 3'end because, they were 5'phosphorylated and 3'blocked. Double strand tester cDNA was prepared by low cycling PCR using tester3 primer and phosphorylated tester5 primer (CAGTGGTATCAACGCAGAGTGGCCATTACGGCCTAGTTACGGG) which is complementary to the 5'tester adaptor. The sense strand of the double strand tester cDNA which is phosphorylated at the 5'end was destroyed by treating it with lambda exonuclease. As a result only the anti-sense strands are retained. Double strand driver cDNA was prepared by low cycling PCR using phosphorylated driver3 primer and driver5 primer (TCCTAAGGTAGCGAGGCCATTACGGCCGGG) which is complementary to the 5'driver adaptor. The anti-sense strand of the double strand driver cDNA which is phosphorylated at the 5'end was destroyed by treating it with lambda exonuclease. As a result only the sense strands are retained.

Anti-sense strands from tester cDNA and sense strands from driver cDNA were mixed and hybridized in 1× hybridization buffer (50 mM Tris-HCl pH 8.0, 0.5 M NaCl, 0.2 mM EDTA) at 68°C for 6 h. The double strand hybrids formed during hybridization were removed by hydroxyapatite chromatography [59] for normalization of the single strand cDNA population. The normalized single strand cDNAs were converted to double strand cDNA and amplified by Failsafe^TM ^PCR system (Epicentre Biotechnologies, USA) using amplification primer (CAGTGGTATCAACGCAGAGT) for which the binding sites are present both in the 5' and 3' end of the cDNA and it is specific to the tester cDNA only (underlined in tester3 and tester5 primers used for cDNA synthesis). The amplified cDNA was digested with *Sfi*I restriction enzyme and cloned in modified pBluescript II SK^- ^in which SfiA (GGCCATTACGGCC) and SfiB (GGCCGAGGCGGCC) sites were introduced between *Eco*RI and *Xho*I. The ligated plasmids were transferred to *E. coli *DH10B-T1^R ^(Invitrogen, USA) to construct the cDNA library.

### Normalization Efficiency

Normalization efficiency was strictly monitored by performing parallel normalization experiment. Chloramphenicol resistance gene with same adaptor and primer sequences was used as reporter gene. In the parallel normalization, the reporter gene was added to the cDNA population as internal control at 1.0% redundant rate. Cloning of cDNA using modified pBluescript II SK^- ^was performed. Normalization efficiency was determined by plating the cDNA library on LB plates containing chloramphenicol.

### Quality Control of cDNA Library

Ninety six colonies from the cDNA library were randomly selected for quality control experiment. Cells from these 96 colonies were used for colony PCR as well as plasmid DNA isolation for sequencing. Colony PCR was performed using M13 forward (GTAAAACGACGGCCAGT) and M13 reverse primer (CAGGAAACAGCTATGAC). The PCR products were run on 1.0% agarose gel with 1.0 kb ladder as marker (Genie, Bangalore, India). Clones without cDNAs (empty clones) will give PCR product size of 226 bp and clones with cDNA will give variable PCR product size higher than 226 bp depending on the length of the insert. Based on this, approximate size of cDNA in each clone was determined. For plasmid DNA isolation, the cells were inoculated in 5 ml LB Broth supplemented with 50 mg/L ampicillin and incubated at 37°C at 200 rpm for 16 hours. Plasmid DNA was isolated from 1.5 ml of culture using Plasmid Miniprep Kit following manufacturer's protocol (Biobasic Inc, Canada). About 200 ng of plasmid DNA was used for sequencing using M13 reverse primer and BigDye^TM ^Terminator v3.1 Cycle Sequencing Kit in 3130xl Genetic Analyzer (Applied Biosystems, CA, USA). These sequences were annotated by using BLASTX algorithm and non-redundant database at NCBI [61].

### Sequencing of Expressed Sequence Tags (ESTs)

The cDNA library was serially diluted and plated on Luria Bertani (LB) agar plates supplemented with 50 μg/ml ampicillin and incubated for 16 hours at 37°C. Well separated single colonies were randomly selected and patched on LB agar plates supplemented with 50 μg/ml ampicillin, and incubated overnight at 37°C. The cells from patched colonies were inoculated in 5 ml LB Broth supplemented with 50 mg/L ampicillin and incubated at 37°C at 200 rpm for 16 hours. Plasmid DNA was isolated from 1.5 ml of culture using Plasmid Miniprep Kit following manufacturer's protocol (Biobasic Inc, Canada). Plasmid DNA was isolated from 12,810 cDNA clones. Sequencing of ESTs was performed using M13 reverse primer and BigDye^TM ^Terminator v3.1 Cycle Sequencing Kit in 3130xl Genetic Analyzer (Applied Biosystems, CA, USA). The raw sequence data was base called by Phred program [62] using DNA Sequencing Analysis Software Version 5.1 (Applied Biosystems, CA, USA). Only the bases having Phred value above 20 were considered for further analysis. The vector sequences and cDNA anchor sequences were removed using Codon Code Aligner (Codon Code Corporation, MA, USA). The clones having less than 100 bp good quality sequences were also removed from the analysis.

### Contigs assembly

Contig assembly of ESTs was done using CAP3 program [63] and Codon Code Aligner (Codon Code Corporation, MA, USA). The default parameters set by CAP3 was used to assemble the ESTs in to contigs and singletons. The number of ESTs in the contigs was used to find the redundancy rate of the ESTs in the library. After the assembly result, the outputs were manually checked and redundant clones were removed. The total numbers of unique ESTs (unigenes) were calculated by adding all singletons and one EST from each contig.

### Annotation of Unigenes

The unigenes were annotated using BLASTX and non-redundant database at NCBI [61]. All the sequences which showed significant similarity (e-value < e^-10^) were assigned putative functions. Others were classified as unigenes with no significant similarity.

### Phylogenetic analysis

Ten ESTs of *J. curcas *from the current study were selected and corresponding genes from five oilseed crops and *Vitis vinifera *were obtained from Genbank at NCBI. The Phylogenetic analysis was done using ClustalW2 program [64].

### Identification of full-length unigenes

Full-length nature of the cDNAs at the 5'end was analyzed from BLASTX output. A unigene was considered as potential full-length gene if (1) there was a *bonafide *5'UTR, (2) the first amino acid in one of the positive reading frames of query sequence matches with the first amino acid in subject sequence from the database and (3) the reading frame covers the entire length of the query sequence. The unigenes that did not show significant similarity were analyzed for Open Reading Frames (ORF) using ORF Finder at NCBI [65]. The unigenes were considered as potential full-length gene if there was a *bonafide *5' UTR and the longest ORF covered almost the entire length of the sequence.

### Functional Classification of Unigenes

The unigenes were annotated again using BLASTN and NT database of arabidopsis at The Arabidopsis Information Resource (TAIR) [66]. This resulted in locus identifier for each unigenes. These locus identifiers were submitted for functional classification using GO annotation tool (GOSlim) at TAIR. Functional classification was done under three Gene Ontology categories viz., cellular component, molecular function and biological process. These three broad categories were further classified with different GO Slim terms.

### Semi-quantitative RT-PCR

Seventeen ESTs for oil biosynthesis genes and one EST for actin gene were selected from the current study and sequenced from 3' ends. Using these 3' end sequences, primers were synthesized to amplify the 3' UTR excluding the poly-(A) tail for all the genes except actin. For actin, the primers were synthesised to amplify a 590 bp fragment which included 392 bp from the 3' end of the coding region and 198 bp from the 3' UTR. The primer sequences and the size of the amplified fragments are given in Table 3. For RT-PCR, total RNA from roots, mature leaves, flowers and developing seeds were treated with DNase and purified using RNeasy Mini Kit following the manufacturer's protocol (Qiagen, Hilden, Germany). About 3.0 μg of purified total RNA from each sample was used for first strand cDNA synthesis using 50 pmol oliog-dT(18) primer and 100 units of PrimeScript^TM ^reverse transcriptase (Takara Bio Inc, Shiga, Japan). Equal quantity of first strand cDNA (from 25 ng total RNA) was used for PCR. Semi-quantitative analysis of the PCR amplified fragments was done by agarose gel electrophoresis and ethidium bromide staining.

**Table 3:** Primers and PCR product size of the genes used for semi-quantitative RT-PCR analysis

S.No	Gene Name	Forward primer	Reverse primer	Product size (bp)
1.	Carboxyl transferase of ACCase β subunit (CT-β)	GAATGAGTTACTTCAGCTTCAC	AATTCACCCTTCTTTCTTGTTGA	307
2.	Biotin carboxyl carrier protein I of ACCase (BCCPI)	GTCGGCTAATCTTAAAGCTATTC	TGTTTATAGCTTCACTAGTGTAC	258
3.	Biotin carboxyl carrier protein II of ACCase (BCCPII)	GATGCTCTCATTGCAATTCTC	TAATGATAAATAACAATGAAGAAGG	278
4.	Malonyl-CoA:ACP transacylase (MCAT)	ACTTCTCCTGTTCAATGGGAA	TCTCTAGTACATGACGCTAATC	314
5.	3-keto acyl ACP Reductase (KAR)	GTTATCTCTCCCGAAAGTGTA	CACAGTATCTGTCACCTTTTC	306
6.	Beta-keto acyl ACP synthase I (KASI)	GCATCTGGCTTGTCTCCAT	GCAGAAGAACACAATTTTGATAC	402
7.	Beta-keto acyl ACP synthase II (KASII)	TGTTCCCAATTTGAAGAAGCAG	AGGAACACCAAATCCAATCTTAT	307
8.	Acyl carrier Protein (ACP)	AAGATCAGTCTAGGAAATCCTTC	GCATAAATTAGGAAATTTTAGAGTGT	180
9.	Acyl ACP thioesterase (FATA)	AATAATGTAGATTTCTTTATTTGTGT	AGTAAACGTAAAACAATACAGTTGAT	285
10.	ω-3-fatty acid Desaturase (ω-3-FAD)	AAAGCTGCAAAATTTTTTATCTGCA	CCCTCTCAAATCCAATCCAA	215
11.	Acyl -CoA binding protein (ACBP)	TGAACATTTCTATGCCGCTTG	GAGAAATAAGGGTCACCATTATC	310
12.	Long chain acyl-CoA synthetase (LCACS)	ATCCATAGCATTTGGCTTTCAAA	CGTGTAGAAGATGAATTGTATAAC	326
13.	Acyl-CoA thioesterase (ACT)	ACTCATCTTAGCTTTGTTATGTTC	CCCATTTCAATCACCGTTTC	173
14.	Glycerol-3-phosphate acyl transferase (GPAT)	GGGTAACGTGTTGGGATTTG	CACAGAATCCAAATAATTCTACATTT	200
15.	Lysophosphatidic acid acyl transferase (LPAT)	TGGTGTATGTTTGTGCTTGG	CTGTACAAAATTGAATCAAGCTTTTT	238
16.	Diacyl glycerol kinase (DGK)	CGGCTATTCGGTTGGAAATAA	GATTTTTGATACAACAAATTACCAGT	296
17.	Triacyl glycerol lipase(TAGL)	CTGGAGACAAGACGAGAATAA	CTTCTATCATAATCAATTATTGTTCG	310
18.	Actin	CAAGTCATCACCATTGGAGCA	GCCTCTTAATTTCGGCTTTAACA	590

### Accession numbers

*J. curcas *unigenes were submitted to the dbEST database of Genbank at NCBI with the accession numbers GW874611 to GW881590 and HO004465 to HO004493.

## Authors' contributions

The study was conceived and directed by PM. RNA isolation, construction of normalized cDNA Library, plasmid DNA preparation, automated DNA sequencing, assembly, annotation, validation of ESTs and other bioinformatics analysis were directed by PM and carried out by PN. Transformation and patching clones were done by DK and PN. Plasmid DNA Isolation and annotation was assisted by GG, JP, NS, PAS, and KKS. PM and PN wrote the paper. All authors read and approved the final manuscript.
